# Numerical Simulation Study on the Mechanism of Formation of Apical Aneurysm in Hypertrophic Cardiomyopathy With Midventricular Obstruction

**DOI:** 10.3389/fphys.2021.717717

**Published:** 2021-07-21

**Authors:** Long Deng, Heng Zuo, An Li, Chun Yang, Xueying Huang

**Affiliations:** ^1^Department of Cardiac Surgery, Fuwai Hospital, Chinese Academy of Medical Sciences, Beijing, China; ^2^School of Mathematical Sciences, Sichuan Normal University, Chengdu, China; ^3^School of Mathematical Sciences, Xiamen University, Xiamen, China; ^4^Network Technology Research Institute, China United Network Communications Co., Ltd., Beijing, China; ^5^Department of Mathematics, Worcester Polytechnic Institute, Worcester, MA, United States

**Keywords:** hypertrophic cardiomyopathy, midventricular obstruction, stress, finite element model, strain, left ventricle

## Abstract

Apical aneurysm was observed to be associated with midventricular obstruction (MVO) in hypertrophic cardiomyopathy (HCM). To investigate the genesis of the apical aneurysm, the idealized numerical left ventricular models (finite-element left ventricle models) of the healthy left ventricle, subaortic obstruction, and midventricular obstruction in HCM of left ventricle were created. The mechanical effects in the formation of apical aneurysm were determined by comparing the myofiber stress on the apical wall between these three models (healthy, subaortic obstruction, and midventricular obstruction models). In comparing the subaortic obstruction model and MVO model with HCM, it was found that, at the time of maximum pressure, the maximum value of myofiber stress in MVO model was 75.0% higher than that in the subaortic obstruction model (654.5 kPa vs. 373.9 kPa). The maximum stress on the apex of LV increased 79.9, 69.3, 117.8% than that on the myocardium around the apex in healthy model, subaortic obstruction model, and MVO model, respectively. Our results indicated that high myofiber stress on the apical wall might initiate the formation process of the apical aneurysm.

## Introduction

Hypertrophic cardiomyopathy (HCM) is an inherited myocardial disease defined by unexplained cardiac hypertrophy, which occurs in about 1 of every 500 adults in the general population (Semsarian et al., 2015). The manifestation of HCM disease is mostly dependent on the hypertrophy and/or obstruction at different intracavitary position (Efthimiadis et al., 2013). Midvertricular obstruction (MVO) is the impedance to flow at the middle level of the left ventricle, while subaortic obstruction is caused by hypertrophy in the basal septum (Falicov et al., 1976). It’s been observed that the presence of MVO is more likely to relate with the formation of apical aneurysm (Minami et al., 2011), which do not happen in HCM patients with mere subaortic obstruction. Although HCM with subaortic obstruction has been adequately studied to date, the clinical features of midventricular obstruction and the mechanisms for the formation of apical aneurysm remain undetermined (Harada et al., 2001).

It has been well accepted that the increase in ventricular wall stress will lead to various pathophysiological process in the heart, including hypertrophy, ischemia and fibrosis (Grossman, 1980; Blaauw et al., 2010; Genet et al., 2014; You et al., 2018). Considering the pathological basis of apical aneurysm is myocardial fibrosis, we hypothesized that the increased ventricular wall stress might contribute to the formation of apical aneurysm in MVO. However, the information of the wall stress was lacking with current clinical examinations. A computational ventricular model of the heart that use finite element method may provide the information of ventricular wall stress and strain, which has been adopted to analyze cardiac computational mechanics and becomes increasingly common (Walker et al., 2005; Tang et al., 2016; Yu et al., 2019; Yin et al., 2020). The present study was undertaken to investigate the mechanisms of the formation of apical aneurysm in HCM, based on the numerical simulation results of three constructed idealized finite element models with different positions of myocardial hypertrophy (healthy, subaortic obstruction, and midventricular obstruction). The obtained systolic myocardial stress/strain in these models were compared to determine the mechanical effects of different hypertrophy positions.

## Materials and Methods

### Idealized Geometries

**FIGURE 1:**
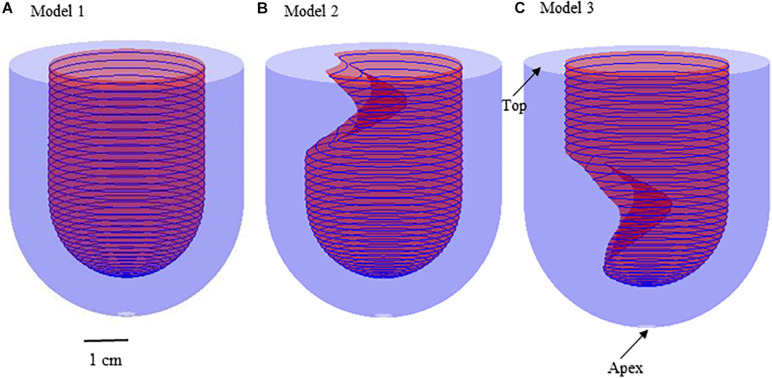
3D Geometries for the construction of three idealized models. **(A)** Model 1: healthy model of left ventricle; **(B)** Model 2: subaortic obstruction of left ventricle in hypertrophic cardiomyopathy; **(C)** Model 3 (MVO model): midventricular obstruction of left ventricle in hypertrophic cardiomyopathy.

**FIGURE 2:**
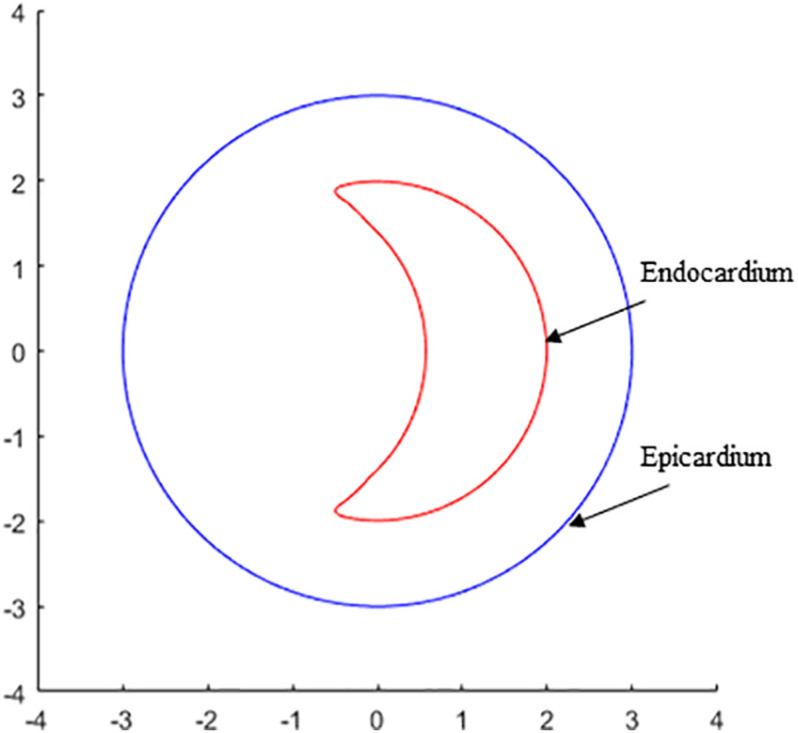
The contour plot of the slice, on which the hypertrophy was most severe, shows the endocardium and epicardium of left ventricle for the constructions for Model 2 and 3.

Three idealized geometries of left ventricle with no obstruction, subaortic obstruction, and midventricular obstruction of hypertrophic cardiomyopathy (Figure 1) were constructed. The geometry of Model 2 and Model 3 were adjusted based on the ellipsoid model (Model 1), which is the idealized model of healthy left ventricle. The mathematical models for inner/outer surfaces (endocardium/epicardium) of Model 1 were given by,

(1) $$\left\{ \begin{array}{c} x=R\sin\theta \cos\varphi \\ y=R\sin\theta \sin\varphi \\ z=\text{R–cos}\pi \end{array}\right.$$

where 0 2, 0 *f*/2. R 2 cm represents the endocardium model; R3 represents the epicardium model. The positions of myocardial hypertrophy were numerically adjusted (Figure 1). Model 2 is the model of subaortic (basal septum) hypertrophy, and Model 3 is the MVO model in which the hypertrophy occurs at the midcavitary level. The figure of the slice, on which the hypertrophy was most severe, for Model 2 and 3 was presented on Figure 2.

### Finite Element Modeling

#### Solid Model

The material of LV was assumed to be hyperelastic, isotropic, nearly incompressible, and homogeneous. The governing equations for the structure models are (summation convention is used) given as follows,

(2) $$\rho v_{i,tt}=\sigma_{ij,j},i,j=1,2,3\left(EquationofMotion\right)$$

(3) $$\varepsilon_{ij}=\left(v_{i,j}+{\text{v}}_{j,i}+{\text{v}}_{\alpha ,i}{\text{v}}_{\alpha ,j}\right)/2,i,j,\alpha =1,2,3,$$

where *t* stands for time, *i*, *j* and label spatial coordinates, **v** is the solid displacement vector, **σ = [****σ**_i,j_**]** is the stress tensor, and ρ is material density. *f*_.,j_ stands for derivative with respect to the *j*th variable. Here, body force due to gravity was ignored. The nonlinear Mooney-Rivlin model was used to describe the material properties of the LV material with parameter values chosen to match experimental data available (Sacks and Chuong, 1993; Bathe, 2002; Humphrey, 2002; Tang et al., 2010). The strain energy function for the modified Mooney-Rivlin model is given by Bathe (2002) andTang et al. (2010),

(4) $$W=c_{1}\left(I_{1}-3\right)+c_{2}\left(I_{2}-3\right)+D_{1}\left[\exp\left(D_{2}\left(I_{1}-3\right)\right)-1\right]$$

where *I*_1_ = ∑*C*_ii_, and ${\text{I}}_{2}=\frac{1}{2}\left(I_{1}^{2}-C_{\text{ij}}C_{\text{ij}}\right)$, are the first and second strain invariants, **C** = [*C*_ij_] = **X**^*T*^**X** is the right Cauchy-Green deformation tensor, **X** = [*X*_ij_] = [∂⁡*x*_*i*_/∂⁡*a*_*j*_], where *x_i_* is the current position, *a_i_* is the original position, *n_f_* is the fiber direction, and *c_i_*, and *D_i_* are material constants chosen to match experimental measurements: *c*_1_ = 18.4 kPa, *c*_2_ = 0, *D*_1_ = 7.2 kPa, and *D*_2_ = 2.0 (Bathe, 2002; Yang et al., 2007).

#### Boundary Conditions

The apex of LV was constrained from moving in the circumferential direction, and the top of the LV is fixed. The pressure condition applied here was obtained from our previous patient-specific model (Deng et al., 2018). The pulsating pressure conditions were uniformly imposed at LV endocardium wall. The maximum value of pressure (LV end of diastolic pressure) was prescribed to be 10 mmHg and the minimum value (LV end of systolic pressure) was prescribed to be 180 mmHg in all models.

#### Active Model

It’s been verified that the results of wall stress in active models were significant different with those obtained from passive model (Huang et al., 2021). To be realistic, the active model was employed in this study for more accurate simulation of cardiac biomechanics. Since the models were idealized model, the simulation started from the zero-load state. After the inlet pressure increased, the LV expanded in both circumferential axis and longitudinal axis directions. In our model, the active tension was implemented by specifying the external forces on epicardium during isovolumic systole phase. The external forces values were obtained by scaling the internal pressure of LV to match the ideal LV volumetric changing trending during one cardiac cycle, which has been verified by clinical measured data (Deng et al., 2018). The real LV active contraction motion was implemented.

#### Mesh Generation and Solution Method

3D tetrahedral (4-node) isoparametric displacement-based finite elements were employed. The finite element model was solved by ADINA (ADINA R&D, Watertown, MA) using unstructured finite elements and the Newton-Raphson iteration method. Mesh analysis was performed for each model by reducing the mesh density in each dimension by 10% until differences between solutions from two consecutive meshes were negligible (less than 1% in L_2_-norm). More details can be found in previously published papers (Yang et al., 2007; Tang et al., 2011).

#### Data Extraction and Statistical Analysis

The stress and strain are all tensors. Therefore, the maximum principal stress (Stress-P_1_) and maximum principal strain (Strain-P_1_) at each node were chosen as wall stress and wall strain, respectively, for convenience. The data for wall stress and wall strain of all integral nodes on the inner boundary of left ventricle (Endocardium) were extracted from the simulation results. The nodes on the region of apex and around apex of LV were selected for investigation. The data was expressed as mean ± SD. Paired *t-*test was used to compare the differences of wall stress/strain between different models. *P* < 0.05 was established as the level of statistical significance. All statistical analysis was performed with R software (version 3.5.1).

## Results

**FIGURE 3:**
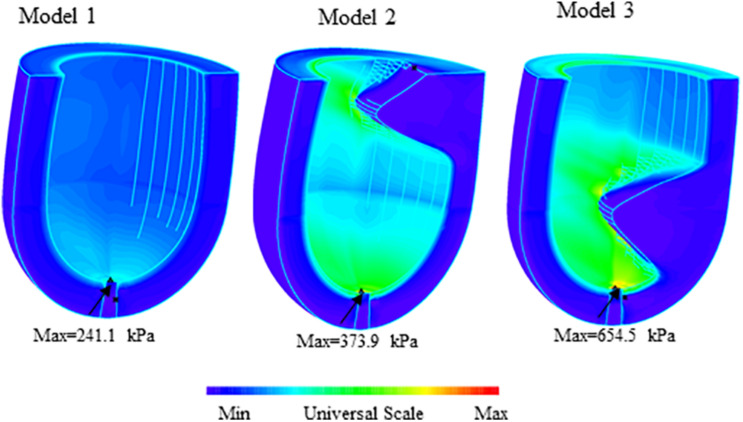
Band plots of wall stress distribution on the left ventricle for Models 1–3. The stress on healthy model (Model 1) was significantly less than other two HCM models. The stress on midventricular obstruction model (Model 3) was significantly higher than that in subaortic obstruction model (Model 2).

An overview of simulation results of wall stress obtained from three models are shown in Figure 3. Table 1 summarized and compared the maximum and average values of stress and strain results on the apex of left ventricle for Models 1–3.

**TABLE 1:** Summary of wall stress and wall strain results in three models.

	Mode1	Model 2	Model 3	Diff (%)
MaxStress (kPa)	241.1	373.9	654.5	75.0
AverageStress (kPa)	67.5	127.4	138.9	9.0
MaxStrain (kPa)	0.243	0.414	0.516	24.7
AverageStrain (kPa)	0.117	0.230	0.273	18.9

*Diff: the difference between MVO model and Subaortic Obstruction Model (base model).*

### Stress in the MVO Model Was Higher Than That in the Subaortic Obstruction Model

**FIGURE 4:**
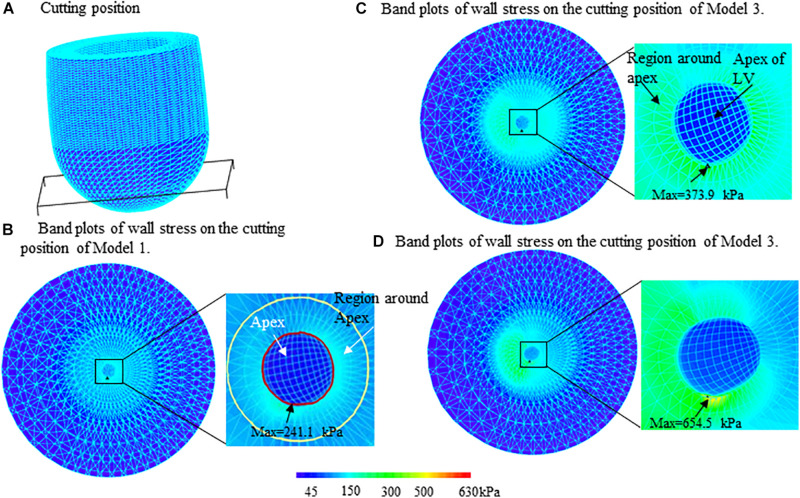
Band plots of wall stress on the apex and regions around apex of left ventricle for three models. The stress on both of the apex and region around apex in midventricular obstruction (MVO) model (Model 3) were significantly higher than that in Models 1 and 2. **(A)** Cutting position. **(B)** Band plots of wall stress on the cutting position of Model 1. **(C)** Band plots of wall stress on the cutting position of Model 2. **(D)** Band plots of wall stress on the cutting position of Model 3.

Figure 4 presents the band plots of stress on the cutting position (Figure 4A) for each models. The stress distribution on the apex of LV was enlarged to present more details. It was found that the maximum stress value on the apex of LV in Model 3 (MVO model) is 75.05% higher than that in subaortic obstruction model (654.5 kPa vs. 373.9 kPa). The average value of stress on the apex area of LV in Model 3 is 138.9 kPa, which is slightly higher than that in Model 2 (127.4 kPa, 9% increase). The wall stress in Model 3 on the apex of LV was statistically significantly higher than that in Model 2 (p = 0.014).

### Stress Level on the Apex of LV Was Significantly Higher Than That on the Other Part of LV

The comparisons of maximum stress on the apex of LV and myocardium around the apex (Figure 4) for three models were summarized on Table 2. It was found that the maximum wall stress on apex was 79.9, 69.3, and 117.8% higher than that on the region around apex for Models 1–3, respectively. In MVO model, the maximum stress on apex increased most significantly comparing to that on the region round apex (654.5 kPa vs. 300.5 kPa).

**TABLE 2:** Comparisons of maximum wall stress on apex and the region around apex of LV for Models 1–3.

	Maximum Stress		
	Apex (kPa)	AroundApex (kPa)	Diff (%)
Model 1	241.1	134.0	79.9
Model 2	373.9	220.8	69.3
Model 3	654.5	300.5	117.8

### Strain in MVO Model Was Slightly Higher Than That in Subaortic Obstruction Model

The strain value on the apex of LV in MVO model (Model 3) was found slightly higher than that in subaortic obstruction model (Model 2) (0.230=0.034 vs. 0.273=0.090, p < 0.01). The maximum strain value on the apex in Model 3 was 0.516, which is 24.7% higher than that in Model 2 (0.414).

### Stress/Strain in the Healthy Model Was Significantly Less Than That in Patients’ Models

The maximum stress value on the apex for the healthy model (Model 1) was significantly less than subaortic model (241.1 kPa vs. 373.9 kPa) and MVO model (241.1 kPa vs. 654.5 kPa). The maximum stress value on the region around apex of LV in Model 1 (134.0 kPa) is also significantly less than Model 2 (220.9 kPa, 39.3% decrease) and Model 3 (300.5 kPa, 55.4% decrease). The maximum strain on the apex of LV was found 49.1% less than that in the subaortic obstruction model (0.117 vs. 0.230) and 57.1% less than that in MVO model (0.117 vs. 0.273).

## Discussion

In this study, three idealized finite element models, in which, one is the healthy model, the other two are patients’ models with different positions of myocardial hypertrophy, were constructed to investigate the potential mechanical mechanisms of the formation of LV apical aneurysm in HCM patients with MVO. Our results indicated that the wall stress on the apex of LV in the MVO model was significantly higher than that in the subaortic obstruction model and healthy model. This may explain the reason that the apical aneurysm was only found in HCM patients with MVO.

Interestingly, the maximum wall stress on the apex in MVO model was 280 kPa higher than that in subaortic obstruction model (75.0% higher); while the average value of the wall stress on the apex in MVO model is slightly higher (9% higher). This is because the myocardial hypertrophy in midcavitary position might affect only a small region of apex of LV. Therefore, the critical (maximum) stress was believed to be responsible for the formation of the apical aneurysm. Comparing to the healthy model, the maximum stress on the apex in subaortic obstruction model is also significantly higher (55.1%) than that in healthy model; the maximum strain on the apex is 96.6% higher. These results suggested that the subaortic obstruction in HCM may also cause abnormal stress distributions on endocardium of LV. Future work is needed to validate these findings.

Midventricular obstruction (MVO) is a less common phenotype of HCM, it occurs in approximately 10% of entire HCM patients (Elliott et al., 2014). Compared to subaortic obstruction, patients with MVO have worse clinical prognosis, which is believed to be associated with apical aneurysm (AA) (Minami et al., 2011; Osawa et al., 2011; Efthimiadis et al., 2013). AA could lead to ventricular arrhythmias and thromboembolic events, and eventually compromises ventricular function. In cardiovascular diseases, AA is mainly seen in patients suffering myocardial infarction, and its pathological basis is myocardial fibrosis (Friedman and Dunn, 1995). However, in HCM patients without coronary artery lesions, the mechanisms of the formation of AA remain unclear. Our results in this research indicated that, compared to subaortic obstruction, MVO has a significantly increased wall stress in the apex. The increased wall stress could cause cardiomyocytes injury, microcirculation disturbance, myocardial ischemia, and eventually myocardial fibrosis (Di Napoli et al., 2003; You et al., 2018), which might be the potential mechanisms of the formation of AA in HCM. With further validation of patient-specific simulations, we may advocate that patients with MVO should receive surgery (septal myectomy) as soon as possible to decrease the wall stress, and thereby prevent the occurrence and development of AA to avoid the associated poor prognosis.

Instead of patient-specific models, the idealized models were employed in this research. The reasons were as follows, (1) Midventricular obstruction appear alone in HCM is very rare, the patients have MVO in HCM in LV usually combined with subaortic obstruction. It’s hard to obtained patient-specific data of MVO in HCM patients; (2) the geometries and hemodynamic status between different patients for MVO or subaortic obstruction patients may be quite different. The idealized models may identify the position of hypertrophy as an independent factor of the genesis of apical aneurysm.

Several improvements can be added to our models: (a) fluid-structure Interactions models (FSI) models; although the stress may be the most important factor contributes to the formation of AA, the fluid shear stress probably may contribute too. This research was the first step to investigate the mechanisms of apical aneurysm by applying finite element model method. In the future, we will apply the FSI models and investigate hemodynamic effects of the blood flow. (b) Fiber orientation and anisotropic models; the multi-layer anisotropic models may be introduced to make possible improvement in computational prediction accuracies. However, the irregular, disorganized alignment of muscle cells or myocardial disarray was found normally in the heart in HCM patients. The fiber orientation data was not available; (c) patient-specific model will be considered in the future; this is the preliminary study to demonstrate the mechanical effects of the position of hypertrophy; (d) LV remodeling approaches; Myocyte hypertrophy and myocardial fibrosis are all associated with LV remodeling (Türkoðlu et al., 2016). Therefore, it is worth adding the remodeling approached to investigate the mechanism of the formation of apical aneurysms in our future studies. (e) Follow up research should be included when patients’ data are available. Although the stress in subaortic obstruction model is on the higher stress level comparing to healthy model, the AA was not found in HCM patients with subaortic obstruction. AA was only found in HCM patients with midventricular obstruction, in which the stress on the apex of LV was on the higher level comparing to subaortic obstruction model. It would be an interesting topic if there was a threshold of stress level for the formation of AA. The follow up research might help us better understand the mechanisms.

## Conclusion

To investigate the mechanisms of formation of apical aneurysm in HCM, three idealized finite element models were proposed to compare the stress/strain on the apex of LV for healthy patient and patients with different position of myocardial hypertrophy (subaortic and midventricular obstruction in HCM patients). The idealized model may help us identify the position of hypertrophy as an independent factor of the genesis of apical aneurysm. It was found that the stress/strain on the apex of LV in midvertricular obstruction model was significantly higher than that on both the subaortic obstruction model and the healthy model. The obtained results suggested that the midventricular obstruction significantly increase the myofiber stress in the apex of LV, which might directly initiates the apical aneurysm. These results are preliminary studies, and the related patient-specific model studies and follow up studies in the future may help us better understand the association between midventricular obstruction and apical aneurysm.

## Data Availability Statement

The original contributions presented in the study are included in the article/supplementary material, further inquiries can be directed to the corresponding author/s.

## Author Contributions

LD and XH designed the study. HZ, XH, and CY constructed the models and performed the numerical simulation. HZ and XH performed the statistical analysis of the data. LD, AL, and XH wrote the manuscript. All authors contributed to the article and approved the submitted version.

## Conflict of Interest

CY was employed by company China United Network Communications Co., Ltd. The remaining authors declare that the research was conducted in the absence of any commercial or financial relationships that could be construed as a potential conflict of interest.
